# Revolutionising Acute Cardiac Care With Artificial Intelligence: Opportunities and Challenges

**DOI:** 10.1016/j.cjca.2024.06.011

**Published:** 2024-06-18

**Authors:** Gemina Doolub, Shaan Khurshid, Pascal Theriault-Lauzier, Alexis Nolin Lapalme, Olivier Tastet, Derek So, Elodie Labrecque Langlais, Denis Cobin, Robert Avram

**Affiliations:** a Department of Medicine, Montréal Heart Institute, Université de Montréal, Montréal, Québec, Canada; b Cardiovascular Disease Initiative, Broad Institute of MIT and Harvard, Cambridge, Massachusetts, USA; c Demoulas Center for Cardiac Arrhythmias, Massachusetts General Hospital, Boston, Massachusetts, USA; d Division of Cardiovascular Medicine, Stanford School of Medicine, Palo Alto, California, USA; e Heartwise (heartwise.ai), Montréal Heart Institute, Montréal, Québec, Canada; f Mila—Québec AI Institute, Montréal, Québec, Canada; g University of Ottawa, Heart Institute, Ottawa, Ontario, Canada; h Polytechnique Montréal, Montréal, Québec, Canada

## Abstract

This article reviews the application of artificial intelligence (AI) in acute cardiac care, highlighting its potential to transform patient outcomes in the face of the global burden of cardiovascular diseases. It explores how AI algorithms can rapidly and accurately process data for the prediction and diagnosis of acute cardiac conditions. The review examines AI’s impact on patient health across various diagnostic tools such as echocardiography, electrocardiography, coronary angiography, cardiac computed tomography, and magnetic resonance imaging, discusses the regulatory landscape for AI in health care, and categorises AI algorithms by their risk levels. Furthermore, it addresses the challenges of data quality, generalisability, bias, transparency, and regulatory considerations, underscoring the necessity for inclusive data and robust validation processes. The review concludes with future perspectives on integrating AI into clinical workflows and the ongoing need for research, regulation, and innovation to harness AI’s full potential in improving acute cardiac care.

## RÉSUMÉ

Cet article examine l’application de l’intelligence artificielle (IA) aux soins cardiaques aigus, en soulignant son potentiel de transformation des pronostics pour les patients face au fardeau mondial des maladies cardiovasculaires. Nous explorons la manière dont les algorithmes d’IA peuvent traiter rapidement et précisément les données pour la prédiction et le diagnostic des affections cardiaques aiguës. Cette revue examine l’impact de l’IA sur la santé des patients à travers divers outils de diagnostic tels que l’échocardiographie, l’électrocardiographie, la coronarographie, la tomodensitométrie cardiaque et l’imagerie par résonance magnétique, discute du paysage réglementaire de l’IA dans les soins de santé et classe les algorithmes d’IA en fonction de leur niveau de risque. En outre, elle aborde les défis de la qualité des données, de la généralisabilité, des biais, de la transparence et des considérations réglementaires, en soulignant la nécessité de disposer de données exhaustives et de processus de validation solides. L’étude se termine par la description des perspectives d’avenir sur l’intégration de l’IA dans les flux de travail cliniques et sur le besoin permanent de recherche, de réglementation et d’innovation pour exploiter tout le potentiel de l’IA dans l’amélioration des soins cardiaques aigus.

Cardiovascular diseases (CVDs) continue to pose a substantial global health burden, accounting for substantial morbidity and mortality worldwide. According to the World Health Organisation,^1^ CVDs are the leading cause of death globally, accounting for an estimated 17.9 million each year, 32% of all global deaths. Within the broad spectrum of cardiovascular health, acute cardiac conditions, such as acute myocardial infarction (AMI), arrhythmias, and heart failure, demand prompt and accurate diagnosis and intervention.

It is within this challenging backdrop that the transformative potential of artificial intelligence (AI) emerges as a promising tool. With its capacity to process vast data sets, recognise complex patterns, and make data-driven predictions, AI has the potential to revolutionise acute cardiac care.^2,3^ The distinction between AI and machine learning (ML) is crucial here: AI refers to the broader concept of machines performing tasks in ways that mimic human intelligence, encompassing reasoning, learning, and problem solving. ML is specifically about algorithms that learn from data to make predictions or decisions. Deep learning (DL), a more advanced subset of ML, involves neural networks with multiple layers that can learn from vast amounts of data at a granular level. By harnessing the power of ML algorithms, AI may expedite the diagnosis of acute cardiac conditions, enhance the accuracy of prognostic assessments, and ultimately improve patient outcomes.^4,5^

**Figure 1.**
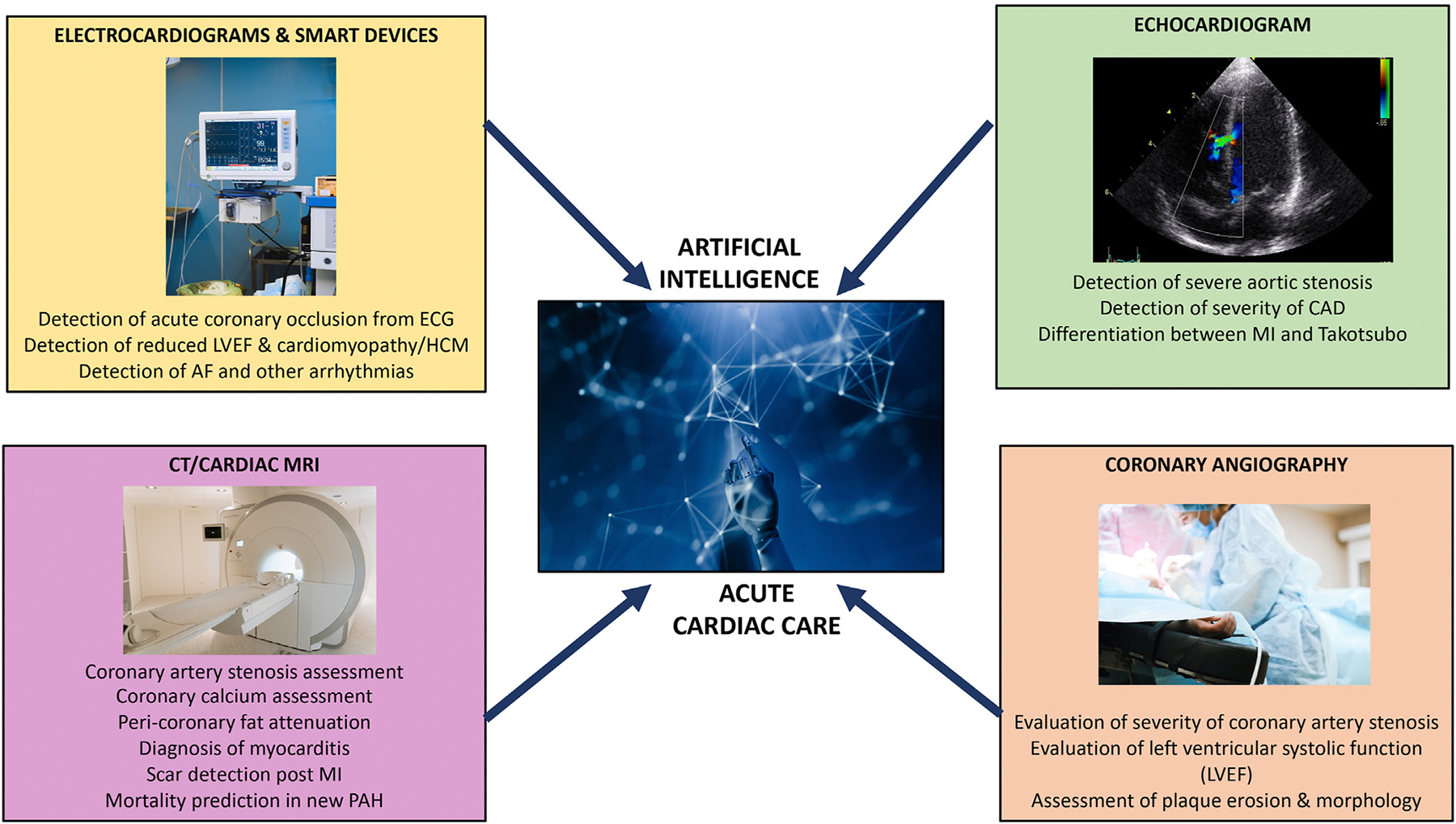
Current applications of artificial intelligence in acute care. AF, atrial fibrillation; CAD, coronary artery disease; ECG, electrocardiogram; HCM, hypertrophic cardiomyopathy; LVEF, left ventricular ejection fraction; MI, myocardial infarction; PAH, pulmonary arterial hypertension.

This state-of-the-art review extends beyond previous examinations of AI in cardiovascular care^3,6–8^ by offering a comprehensive overview of current AI tools specifically tailored for acute cardiac care (Fig. 1). It explains the influence of AI on clinical outcomes and operational efficiency, outlines pertinent regulatory frameworks pertinent to bringing these tools to clinical practice, and offers insights to inform the trajectory of future developments in this rapidly evolving field. Appropriate peer-reviewed papers and articles were hand-selected by the authors of this review according to their relevance, accuracy, and expertise level, as well as their conveying novel, contemporary, and up-to-date information.

## AI Algorithms as Medical Devices

Before describing actual AI applications, it is important to provide some overview on how AI devices are approved and regulated. Health Canada’s medical device classification^9^ system describes 4 classes of devices according to the inherent risk to public health. Class I devices do not require Health Canada approval and represent low-risk devices. Class IV devices are usually devices that are implanted surgically, such as pacemakers. AI algorithms usually fall under class II and class III, each defined by their potential risk on patient health. Class II devices are those where AI is used to support cardiac care, such as in monitoring heart rate or analysing electrocardiographic (ECG) data for noncritical conditions, where an erroneous result does not lead to immediate danger for the patient. The impact on patient health escalates with class III devices, which are used in critical cardiac care scenarios, such as the real-time diagnosis and management of acute cardiac events such as AMI. For class III devices, the accuracy of AI is crucial, because erroneous readings or a missed alert could lead to catastrophic outcomes, including death, long-term disability, or other serious morbidity. Although the AI applications mentioned in this review are not approved by Health Canada yet, whether they fall under either class II or class III will depend on how serious the cardiac condition is and how much the clinicians rely on the application’s output for decision making.

In the United States, approval of AI devices by regulatory bodies was expected to rise 30% in 2023 compared with the previous year.^10^ In the Food and Drug Administration’s (FDA) regulatory scheme, software is subject to specific criteria under which it is considered a medical device. Software as a Medical Device (SaMD) encompasses software that can perform one or more medical functions without being part of a hardware medical device, as is often the case with AI algorithms. The FDA’s guidance for clinical decision support software^11^ provides clarity on when such software is regarded as a medical device. Conversely, software is a medical device when it involves more active functionalities, such as processing signals or images, providing time-critical outputs, or generating risk scores for diseases. This distinction is particularly pertinent in cardiology, where AI-driven SaMD might analyse ECG waveforms or assist in the detection and diagnosis of cardiovascular conditions. In such instances, SaMD is crucial for decision making in clinical settings, and accuracy is paramount because erroneous recommendations or missed alerts could have severe consequences for patient health. The FDA’s Breakthrough Devices Program^12^ aims to fast-track the development and review of SaMDs critical for treating life-threatening or irreversibly debilitating conditions. It extends to devices addressing health disparities and nonaddictive treatments for pain or addiction. While accelerating patient access to breakthrough technologies, the program maintains the FDA’s high standards for premarket approval, 510(k) clearance, and *de novo* marketing authorisation. The majority of FDA-approved AI-enabled devices or SaMDs^10^ were approved in the past year through the 510(k) pathway in cardiology.^13^

## Deployment of AI in Acute Cardiac Care

AI has the potential to extract complex features from images and data sets to make accurate predictions. AI’s capability extends to identifying subtle, imperceptible patterns that escape human detection, such as predicting left ventricular ejection fraction (LVEF) from ECGs^14,15^ and coronary angiograms.^16,17^ In the context of acute cardiac care, the integration of AI presents a promising avenue for predicting events, thus enhancing patient outcomes and health care efficiency. However, for AI to effectively operate in this critical domain, several key prerequisites must be met. First, quick turnaround is paramount as real-time decision-making is imperative in acute cardiac situations. ML may offer a significant benefit owing to its ability to process and interpret large data sets far more quickly than human analysis to guide real-time decisions. Second is the capacity for AI to report erroneous predictions, helping health care providers scrutinise and rectify any inaccuracies and maintain a log to prevent the replication of similar errors in the future, maintaining a high standard of patient safety. Third, fairness in algorithms^18^ is crucial to prevent biases that may disproportionately affect certain patient groups (eg, women, ethnic minorities), thus ensuring equitable access to accurate care. In addition, there should be comprehensive training for health professionals on how to use and interpret these algorithms to avoid user bias, misuse, or overreliance on the algorithm that would potentially compromise patient care. Fourth, akin to nutrition labels on food products, AI in acute cardiac care should provide a transparent label, offering health professionals insights into the AI application’s data sources, model accuracy, and potential limitations.^19^ These fundamental considerations lay the foundation for the responsible and effective utilisation of AI in acute cardiac care, ultimately improving patient outcomes and bolstering the quality of emergency medical services.

## Current Applications in Acute Cardiac Care

### Electrocardiography

The incorporation of AI into ECG has the potential to benefit for both patients and health care providers in clinical practice, offering patients rapid and precise diagnoses and enabling prompt and effective interventions crucial in acute cardiac scenarios, such as ventricular arrhythmias and MI (Table 1). One such example is the occlusion myocardial infarction AI ECG model developed by Herman et al.,^20^ which was developed using > 18,000 ECGs from patients with suspected acute coronary syndrome (ACS). The primary outcome was acute coronary occlusion (ie, a 100% acutely blocked coronary) requiring urgent percutaneous coronary intervention. The model achieved an area under the receiver operating characteristic curve (AUC) of 0.938 (95% confidence interval [CI] 0.924–0.951), with superior performance compared with the currently used ST-segment elevation MI criteria and similar performance compared with expert ECG readers^20^ (accuracy 90.9% [95% CI 89.7%–92.0%], sensitivity 80.6%). Similarly, Al-Zaiti et al.^21^ implemented an ML algorithm for risk stratification of occlusion MI, yielding an AUC of 0.79, sensitivity of 68.2%, and a negative predictive value of 92.5%.^22^ In addition to increasing yield for true cardiac events, AI-driven ECG analysis could reduce false-positive results, minimising unnecessary anxiety and additional testing for patients. For example, Fiorina et al.^23^ used DL to predict life-threatening ventricular arrhythmias, achieving an AUC of 0.911, with high sensitivity (83.3%) and specificity (88.7%). Finally, in the context of arrhythmias, the FDA recently approved the Irregular Rhythm Notification Feature.^24^ This software-only mobile application, designed for use with a smartwatch, analyses pulse rate data to detect episodes of irregular heart rhythms, potentially indicating atrial fibrillation. When such episodes are detected, the application sends a notification to the user.

Furthermore, the use of AI to analyse ECGs could enhance physicians’ diagnostic capabilities in the future. This technology may allow for the detection of a wider range of conditions beyond the traditional scope of ECG analysis, enabling the identification of diseases that cardiologists would not typically detect with the use of standard ECG methods. For example, AI models such as the DeepECG-HFrEF,^22^ an AI-ECG convolutional neural network (CNN),^26^ and a CNN by Attia et al.^28^ have demonstrated utility in detecting conditions such as heart failure with reduced ejection fraction (LVEF < 40%; AUC: 0.844), dilated cardiomyopathy (AUC 0.955), and left ventricular systolic dysfunction (LVEF < 35%; AUC 0.918), respectively. Silva et al.^25^ harnessed multiple neural networks (en “ensemble” of algorithms) to diagnose acute pulmonary embolus, with an AUC of 0.75. These studies show the significant potential of AI applications in providing better diagnostic precision and risk evaluation in acute cardiac care.

From a practical point of view, in 2023, the FDA cleared the first 2 AI algorithms designed to analyse ECG data for use in cardiovascular screening.^31^ The first, Anumana ECG-AI (Cambridge, MA),^28,32,33^ uses an algorithm that analyses a standard 12-lead ECG to output the likelihood of reduced LVEF < 40%. The EAGLE study^34^ was a randomized controlled trial in which 120 primary care teams were randomised to have access to AI results or to traditional clinician care. The researchers found that AI intervention increased the diagnosis of low LVEF (≤ 50%) within 90 days of the ECG, indicating that the algorithm based on ECGs could allow for early diagnosis of reduced LVEF in primary care settings.^34^ The second AI tool to be cleared by FDA is the Viz.AI HCM module (San Francisco, CA), which analyses routine 12-lead ECGs taken at the point of care to screen patients at high risk of hypertrophic cardiomyopathy (HCM), notifying cardiologists on a mobile application so that follow-up with echocardiography can be scheduled. ^31,35^

Such innovations are now being integrated into clinical practice. Lin et al. reported the use of AI-interpreted ECGs compared with usual care among patients who came to the emergency department.^36^ The authors showed that the AI model reduced time from ECG to entry in the catheterisation lab (43.3 min vs 52.3 min; *P* = 0.003). Although the study needs to be replicated outside Taiwan to confirm generalisability,^37^ it illustrates the potential benefits of using AI to speed up and simplify the delivery of patients with ST-segment elevation MI to the catheterisation lab, possibly saving time and heart muscle. For example, the Mayo Clinic has developed an AI ECG dashboard, where the medical record of any patient includes access to results of AI-based ECG analysis, including the likelihood of LV systolic dysfunction, silent atrial fibrillation and HCM.^38^

### Echocardiography

The integration of AI into echocardiography in the past few years has resulted in significant developments for acute cardiac care to automate many of the routine measurements and diagnoses that echocardiographers obtain as part of their routine assessment (Table 2). For example, Holste et al.^39^ used a 3D CNN in an effort to detect severe aortic stenosis based on single-view 2-dimensional echocardiography, achieving an AUC of 0.978, with high sensitivity (85%) and specificity (96%). This novel method has the potential to efficiently screen for a condition associated with substantial morbidity and mortality, that is increasing with population aging..^39^ Similarly, Upton et al.^40^ used an automated image processing pipeline to assess the severity of coronary artery disease with the use of stress echocardiography, attaining an AUC of 0.93, with sensitivity of 92.7% and specificity of 84.4%. Deployment of these and related models may shift the initial burden away from trained echocardiographers and core laboratories, enabling more efficient point-of-care screening and diagnostic pathways designed to detect target conditions at their earliest stages.^39^

Laumer et al.^41^ used an autoencoder model for the differentiation between AMI and takotsubo syndrome, demonstrating the potential of AI in distinguishing complex cardiac conditions on transthoracic echocardiography. Takotsubo syndrome is often associated with acute ventricular dysfunction and can lead to malignant arrhythmias and death. Takotsubo syndrome, like AMI, can present with acute chest pain, ECG changes, and abnormal troponins. A prompt diagnosis of takotsubo syndrome is therefore important. In their study, Laumer et al. used apical 2-chamber and 4-chamber echocardiography images of 228 patients (114 with takotsubo syndrome and 114 with AMI) to train their algorithm. A temporal CNN was used to predict the likelihood of takotsubo syndrome and AMI for each patient. A mean AUC of 0.79 and accuracy of 74.8% was obtained for the AI algorithm, compared with an AUC of 0.71 and accuracy of 64.4% for cardiologists, suggesting that the system performed favourably compared with cardiologists. Kusunose et al.^42^ used ResNet-based models to detect wall motion abnormalities in patients admitted with AMI. The algorithm achieved an AUC of 0.99 for detecting the presence of wall motion abnormalities, which was similar to the AUC of 0.98 achieved by cardiologists and sonographers (*P* = 0.15). Importantly, the algorithm’s performance was better than that of residents (AUC 0.90; *P* = 0.002).^42^ Thus, the accuracy of the DL algorithm was superior to that of inexperienced observers and similar to that of experienced sonographers.^42^ This could signal the application of AI in the interpretation of large volumes of echocardiographic images, at the point of care, and in shorter time frames, thus enabling faster and accurate risk stratification among patients admitted with AMI.

Ouyang et al.^43^ used 3-dimensional convolutions to predict heart failure with reduced ejection fraction, highlighting AI’s capacity for prognostication. And Zhang et al.^44^ highlighted AI’s potential in identifying HCM and cardiac amyloidosis with impressive AUC values. These findings underscore the possibilities arising from the use of AI in echocardiography for acute cardiac care, which would include streamlining clinical decision making in patients admitted with different cardiac pathologies. Another important feature is the potential for relatively inexperienced readers to learn from AI models to improve their performance and match experienced readers.

### Coronary angiography

Recent studies have demonstrated AI’s potential impact on various aspects of coronary angiography (Table 3), including the assessment of LVEF and the severity of coronary stenosis. Notably, estimating LVEF according to a coronary angiogram is impossible for cardiologists, highlighting the significance of AI in this context. Avram et al.^16^ developed a video-based deep neural network to evaluate LVEF with the use of left coronary angiograms with an impressive AUC of 0.906, highlighting AI’s potential to expand the current uses of coronary angiography. Although the algorithm tended to overestimate low LVEFs and underestimate high LVEFs, it could confer added clinical utility by providing an early estimate of cardiac function at the time of coronary angiography after an AMI, thus contributing to the prompt identification and management of patients at risk of heart failure and fluid overload at the time of the procedure. In addition, the same team harnessed purpose-built neural networks in the form of an algorithmic pipeline (CathAI), to assess coronary stenosis severity,^45^ achieving an AUC of 0.862. Human expert visual quantification is known to display significant variability,^46^ with studies showing an overestimation of disease severity by visual assessment.^47^ DeepCoro^48^ built on this by incorporating vessel tracking and a video-based Swin3D model that was trained and validated on a large data set consisting of 182,418 coronary angiograms. DeepCoro achieved an accuracy of 71.89% in identifying coronary artery segments, with a classification AUC of 0.830 (95% CI 0.822–0.837) in predicting severe (≥ 70%) stenosis. Compared with 2 expert interventional cardiologists, DeepCoro showed lower variability than the clinical reports. Models like CathAI and DeepCoro could eventually provide a basis for performing automated, standardised, and more reproducible evaluations of coronary disease severity, which would supplement cardiologists’ visual assessment and help in decision making regarding intervention.

AI has also demonstrated its ability in detecting and characterising complex coronary phenomena, such as plaque erosion and lesion morphology, as exemplified by “transformer-based”^61^ DL models (frame-wise AUC of 0.971) and Deep Discern–DNN (AUCs of 0.94 at the frame level and 0.91 at the lesion level).^51,50^ Although plaque rupture is the most frequently cited finding in patients with sudden cardiac death, plaque erosion or calcified nodules also have been implicated as underlying mechanisms contributing to ACS in up to 25% of cases.^62^ Identifying vulnerable and erosive coronary plaques in a timely fashion could significantly affect patient management by identifying these high-risk features in a timely fashion and encouraging physicians to treat these lesions accordingly and initiate secondary prevention with aspirin and high-dose statins in a more aggressive manner. Finally, several recent studies^55–57^ have looked at combining deep learning and intravascular imaging to assess deployment characteristics, such as stent area and stent apposition. Previous intravascular imaging studies^63^ have suggested that suboptimal stent deployment can predict worse clinical outcomes, with evidence highlighting significant reductions in major adverse cardiovascular events and cardiac mortality when using optical coherence tomography or intravascular ultrasound imaging when performing percutaneous coronary interventions.^64–67^ Using AI to provide consistent, real-time measurements of indices such as stent expansion and malapposition would enable a more standardised and rapid means of optimising stent results and thus improving patient outcomes.

These AI-driven advances hove the potential to expedite diagnoses, enhance precision, and improve patient outcomes in acute cardiac care, setting a promising trajectory for the future of coronary angiography. With that in mind, Avram et al completed the CathEF prospective study (NCT05317286), to demonstrate the validity and utility of the CathEF algorithm to estimate LVEF at the point of care during ACS. To achieve this, they developed PACS-AI,^68^ a specialised software designed to seamlessly integrate with the hospital’s image data repository. This enables real-time analysis using an AI model during a patient’s coronary angiography procedure. Such software could be expanded to other algorithms or other modalities to allow real-time AI-assistance for computer vision tasks, such as the ones outlined in this paper.

### Cardiac computed tomography

In recent studies, AI has demonstrated its ability to enhance various aspects of cardiac computed tomography (CT) evaluation (Table 4) compared with conventional methods.^4^ For example, Paul et al.^69^ used a neural network for coronary stenosis assessment, yielding a sensitivity of 93% and specificity of 97%, with the DL model detecting > 50% stenoses with a performance similar to that of senior radiologists.^69^ Similarly, Chen et al.^70^ harnessed a DL model to detect coronary stenosis, achieving an AUC of 0.87. This approach not only demonstrated excellent sensitivity but also offered favourable processing speed (time to analyse all segments per patient by DL model was 0.47 min, vs 29.65 min for the reader model; *P* < 0.001) while maintaining diagnostic accuracy similar to that of intermediate and senior radiologists.^70^ Moreover, the utilisation of AI-based CT fractional flow reserve, as explored by Martin et al.,^71^ enables the functional evaluation of coronary lesions, offering a substantial improvement over traditional anatomic assessments. This refined assessment helps guide treatment decisions, ensuring that interventions are directed toward lesions that are truly causing ischemia.

AI has also demonstrated the potential to extract features relevant for cardiovascular health with the use of imaging scans performed for other purposes. For example, on nongated CT scans, AI may identify patients with incidental findings of coronary calcium (CAC) to initiate primary prevention therapy.^77–79^ For example, Eng et al.^77^ trained a gated coronary CT for CAC scoring that aligned closely with traditional manual scoring, then trained a DL model on internal data and a cohort from the Multi-Ethnic Study of Atherosclerosis (MESA) study, obtaining sensitivities of 71%–94% and positive predictive values of 88%–100%^80^ for the recommended threshold to start statins (CAC ≥ 100). NOTIFY-1^78^ was a randomised quality improvement project whereby patients without an established diagnosis of coronary artery disease were screened for CAC with the use of a DL algorithm on a previous nongated chest CT scan. Among 2113 patients meeting initial inclusion criteria, 424 were thus found to have CAC. The authors concluded that opportunistic CAC screening resulted in a major increase in statin prescription.^78^ Peng et al.^79^ used a DL algorithm to measure incidental DL-CAC on non–ECG-gated chest CTs and found that incidental CAC ≥ 100 correlated with higher risk of all-cause death (hazard ratio 1.51, 95% CI 1.28–1.79), compared with DL-CAC = 0. That study highlighted the potential for AI algorithms in screening patients to guide earlier intervention.

Finally, pericoronary fat attenuation on CT as analysed with the use of AI acts as a radiomic metric of coronary inflammation, serving as a marker of increased cardiac mortality.^81^ Oikonomou et al. developed a new AI tool to predict cardiac risk by assessing the radiomic profile of coronary perivascular adipose tissue.^81^ They trained and validated an ML algorithm with 1575 participants in the SCOT-HEART trial.^82^ This new AI-driven imaging biomarker significantly improved major adverse cardiovascular event prediction beyond traditional risk stratification comprising traditional risk factors, CAC, and vulnerable plaque on CT [Δ C-statistic = 0.126; *P* < 0.001)].

AI also addresses technical issues such as image reconstruction, segmentation, and motion correction. All major vendors now offer AI-based image reconstruction algorithms,^83^ and newer approaches improve spatial resolution of coronary CT angiograms by training on standard acquisition data along with that obtained using ultra-high-resolution scanners.

### Cardiac magnetic resonance imaging

The merging of AI in cardiac magnetic resonance (MRI) has opened up exciting possibilities for the management of acute cardiac conditions (Table 5), enhancing the way we diagnose and treat those conditions. Baessler et al.^84^ used texture analysis to detect myocarditis, achieving an AUC of 0.88 when combining the texture features T2 run-length nonuniformity and grey-level nonuniformity, This AI-based approach enables clinicians to swiftly identify with high sensitivity (89%) and specificity (92%). Ghanbari et al.^85^ utilized a CNN for scar analysis and arrhythmia prediction. By accurately assessing scar tissue, clinicians can better plan ablation procedures to treat arrhythmias, improving patient outcomes and reducing the need for repeated interventions.

Zhang et al.^47^ introduced DL virtual native enhancement for scar assessment in MI. This approach provides a comprehensive view of tissue damage, aiding in the selection of appropriate revascularisation strategies and optimising postinfarction care. Sharifrazi et al.^86^ demonstrated the power of a CNN coupled with K-means clustering in the detection of myocarditis, ensuring timely interventions with an impressive AUC of 0.971. Early detection allows for the prompt initiation of antiinflammatory therapies, preventing disease progression and minimising cardiac damage. Moccia et al.^87^ used local binary patterns and spatiotemporal features for scar detection. This AI-driven method assists in identifying regions of fibrosis or scarring, facilitating targeted treatment plans and reducing the risk of arrhythmias. And Alabed et al.^88^ explored AI’s potential in mortality prediction in newly diagnosed pulmonary arterial hypertension with the use of multilinear principal component analysis. By identifying patients at higher risk of mortality, health care providers may offer more intensive monitoring and personalised treatment strategies.

In addition, AI may also streamline MRI image acquisition. In fact, GE Healthcare revealed in 2023 that the FDA had authorized Sonic DL,^89^ which accelerates MRI acquisition speeds up to 12-fold, with a claimed ability to cut scanning time by 83% compared with current best practice, thus reducing the need for repeated patient breath-holds during image acquisition. In conclusion, AI-enhanced cardiac MRI has the potential to not only speed up diagnoses but also to deliver precise risk assessments and, perhaps in future, even facilitate personalised treatment plans for patients with acute cardiac conditions.

## AI in Risk Prediction

In risk prediction after ACS, AI models may create personalised risk profiles for each ACS patient by considering a large range of variables including demographic data, medical history, biomarkers, genetic factors, and even imaging findings. Traditional scores such as GRACE^90^ and TIMI^91^ are useful but tend to provide a one-size-fits-all approach, and they take into consideration only a limited number of variables, which may not accurately reflect the unique characteristics and risks of each patient. In that context, AI algorithms^92^ have shown the ability to outperform traditional risk scores in terms of predictive accuracy. For example, the Korean DAMI study^81,82^ showed their DL risk stratification model to outperform conventional risk scores in predicting 12-month mortality after AMI. Furthermore, d’Ascenzo et al.^93^ were able to develop the PRAISE score, which comprised clinical (eg, age, sex, diabetes, hypertension, previous MI, etc), therapeutic (eg, medications), angiographic (eg, multivessel disease), and procedural variables (vascular access and percutaneous coronary intervention) and showed reliable discriminative abilities for predicting death, MI, and major bleeding after an ACS.

ML and DL models have also been used in hospital settings to predict patients at risk of cardiac arrest.^94^ For example, Lee et al.^95^ developed an ML-based real time model for predicting in-hospital cardiac arrest in an intensive care unit setting with the use of ECG-based heart rate variability and a light gradient–boosting machine algorithm. That study used a relatively large sample size (~5000 patients), and the model achieved an AUC of 0.881 (95% CI 0.875–0.887). Although it has yet to be validated in large multicentre studies, that study highlighted the potential for AI to use ECG data to predict cardiac arrest, maximising performance by integrating nonlinear heart rate variability measures.^95^

## Patient Monitoring and Extraction of Electronic Records Data Using AI

The application of DL algorithms in cardiovascular monitoring devices holds promise in the delivering of precise and efficient health care. One such breakthrough is the PP-Net DL algorithm,^96,97^ which demonstrates its ability to estimate crucial physiological characteristics, including diastolic blood pressure, systolic blood pressure, and heart rate, all with the use of a single-channel photoplethysmography signal. To test the efficacy of the PP-Net, the algorithm was applied to the MIMIC-II database. This open access database comprises simultaneous recordings of various physiologic signals and parameters from intensive care unit patients, including ECG, photoplethysmography, and arterial blood pressure. The model achieved a normalised mean absolute error of 0.09 mm Hg for diastolic blood pressure, 0.04 mm Hg for systolic blood pressure, and 0.046 beats/min for heart rate across the entire sample population of 1557 critically ill patients.^96^ Thus, models such as PP-Net show promise in terms of estimating multiple physiologic parameters simultaneously with the use of sensors that are noninvasive and convenient detection methods, as opposed to invasive monitoring such as arterial lines. This model could have a possible role in intensive care unit settings in the future, as well as in distant monitoring of elderly or cardiac patients.^97^

In an era when patient vital signs, clinical history, and laboratory data are all documented in electronic medical records, AI has the ability to harness data to develop innovative algorithms aimed at predicting critical events.^98^ For example, Kwon et al.^99^ developed a cardiac arrest algorithm by analysing 3 years’ worth of electronic medical record data,^99^ incorporating variables, eg, blood pressure, pulse rate, respiration rate, and body temperature. The study used a data set of 47,505 ECGs from 25,672 adult patients and achieved an AUC of 0.948 for predicting cardiac arrest within 24 hours.^99^ This milestone research marked the first successful development and verification of a DL algorithm for cardiac arrest prediction through ECG analysis. It underscores the potential of AI in predicting cardiac arrest with the use of diverse ECG formats.^99^

## Challenges

The implementation of AI in acute cardiac care is fraught with various challenges, all necessitating careful consideration. First and foremost, the success of AI algorithms heavily relies on the quality and availability of data. In many health care settings, data may be fragmented, inconsistent, and incomplete, which can affect the accuracy and generalisability of AI models.^100^ Generalisability refers to the ability of an AI system to apply insights gained from one set of data to other diverse data sets effectively, which is crucial to ensuring that AI tools developed in one setting are applicable and reliable across various patient populations and clinical environments. Without high generalisability, an AI model might perform well in the specific context it was trained on but fail to provide accurate predictions or useful insights in other contexts, potentially limiting its usefulness for broader usage.

Ensuring standardised and high-quality data is essential to harness AI’s full potential in acute cardiac care. The quality of data^101^ relies on several factors, namely, accuracy of the information entered, consistency in documenting the data, completeness of the data sets used, freshness (ie, are the data up-to-date or not), and relevance. To ensure that data put into in AI models is of as high quality as realistically possible, several steps can be implemented, including data cleaning, preprocessing, and augmentation.^101^ In addition, there is a need for better and more robust external validation, ideally prospective, within the settings in which models are to be deployed (eg, intensive care unit). Another driver of potential AI malfunction is data set shift,^102^ which can be triggered by multiple factors,^102^ including technology/software changes, changes in population/demographics, changes in clinician/patient behaviour, and differences in disease definition and protocols. It is therefore crucial that clinicians remain vigilant to recognise and mitigate these factors. Furthermore, AI systems may inadvertently perpetuate biases present in the data used for their training. It has been shown that women and people of ethnic minorities are underrepresented in cardiovascular research trials.^56^ If the training data contain demographic or socioeconomic biases, AI algorithms will exhibit similar biases,^103^ potentially leading to disparities in patient care.^104^ Bias mitigation^105^ will occupy a pivotal role in the design of AI-based models, and strategies will encompass steps such as preprocessing data before developing the model, encouraging the model to learn balanced predictions with the use of mathematical integrations, and postprocessing.^106^ In fact, the STANDING Together initiative^107^ aims to improve inclusivity and data diversity by drafting guidance for the representation and reporting of data sets.

The inherent complexity of AI models can make it challenging to understand their decision-making processes. In health care, understanding why an AI system reached a particular conclusion is crucial for clinicians to make informed decisions. Every care should therefore be taken to ensure that AI models are designed keeping transparency and interpretability in mind.^108^ This can be achieved through measures such as clear communication, providing comprehensive information, ensuring auditability, and maintaining thorough records.^108^

To facilitate the development of effective AI applications, 2 principal collaborations are key. First, it is crucial for clinicians to cooperate with nonclinician team players, such as data scientists, researchers, engineers, and software developers, through interdisciplinary collaborations. This allows experts from diverse backgrounds to offer their input on AI technology, as well as to identify potential challenging areas in cardiovascular medicine that could be addressed by AI, and through progression cycles, clinicians would have a safe platform to voice their feedback, real-world insights, and concerns, in order to refine AI tools and algorithms developed by researchers and engineers. Such approaches have been taken by agencies such as the Canadian Institute for Advanced Research^68^ to fund research projects involving medical and AI experts working together to solve the responsible integration of AI in health care. Second, clinicians should be encouraged to engage with the private sector and industry partners to seek opportunities to develop cutting-edge AI technology, but also to advocate for safe and ethical data sharing, as well as to put in place safeguards against the potential misuse of AI technology by third parties.

Another challenge that is likely to arise is the cost of resources associated with implementing AI in acute cardiac care. These costs encompass initial start-up investments in hardware such as high-performance computers with graphic cards, software licenses, and specialised staff including software engineers and data scientists to integrate and maintain these tools with electronic medical records. Our team faced this challenge while conducting the CathEF study,^109^ where we integrated our algorithm into the hospital imaging system using a custom-built software called PACS-AI to predict LVEF at the point of care. The cost of developing and initial maintenance of this software was around $200,000, and it requires ongoing maintenance by the hospital team. Therefore, it is essential to establish sustainable collaborative partnerships with industry players, academic institutions, and governmental agencies to balance costs through funding, grants, and expertise and resource sharing.

Finally, as AI becomes increasingly integrated into health care, regulatory frameworks and ethical considerations become paramount. Adherence to privacy regulations (eg, the Health Insurance Portability and Accountability Act of 1996 in the United States)^110^ to protect fundamental ethical principles such as patient consent, patient privacy, confidentiality, and data security and the responsible use of AI in health care, are essential to ensure patient safety and compliance with existing laws.^111^ In addition, there is a need for standardisation across the models and their operational contexts. Newer versions of deep learning frameworks, different than the ones used during model training, have altered the way images are prepared before AI analysis, resulting in potentially varied predictions even when using identical data and models.^112^ It is critical to establish consistent standards for the computational environment, image preprocessing methods, model versions, and data formats, because these elements can significantly affect AI model predictions and their reliability in clinical decision making.

Addressing these challenges is critical to realizing the potential of AI in acute cardiac care, fostering trust among health professionals, and ensuring that the technology benefits patient outcomes while adhering to ethical and regulatory standards.

## Future Perspectives

Research in this domain has been constrained by a lack of forward-looking trials and assessment of how models perform in real-world clinical settings.^113^ Therefore, more studies describing how to integrate AI models in the clinical workflow and demonstrating how AI affects clinical practice are required to gain a clearer understanding of the benefits of AI in guiding screening processes. For example, Avram et al.^68^ have been working on integrating an AI algorithm into a Picture Archiving and Communication System (PACS), which is a universal data repository of radiologic examinations, by developing PACS-AI. The PACS-AI software, which works smoothly with existing hospital systems, gives instant AI results on radiologic exams, allowing the testing of AI algorithms in the clinical process. This integration helps to simplify the work of cardiologists or radiologists, who often deal with large amounts of images, which can cause diagnostic delays, increased work-related stress, and potential misinterpretation of data. In addition, the PACS-AI framework follows the principles of continuous learning and adaptation, using user feedback to adjust and enhance its diagnostic algorithms over time. This feature solves a major problem in conventional AI models, which may become obsolete as medical knowledge and practices change. Developing tools such as PACS AI is important for advancing the field and enabling the evaluation of AI algorithms in clinical settings.

Furthermore, health technology assessment for AI applications will play a pivotal role in evaluating their clinical effectiveness, patient safety, cost-effectiveness, ethical implications, and societal impact. Such assessments can pose specific challenges, which include the complexity and dynamic fast pace of certain algorithms, the difficulty in accessing high-quality diverse data for training and validation, and the need for AI applications to show solid, reliable performance across a range of patient demographics and clinical situations. It has been argued that that health technology assessments for AI tools must enable shorter device lifespans, in view of the rapidly evolving nature of the technology.

Most medical AI applications in acute care are limited to narrow applications and task-specific models, hindering their widespread adoption. The use of generative AI in acute cardiac care is likely to emerge as a game changer in the future owing to its more versatile approach, allowing for more flexible learning and diverse interpretation of medical images. The use of generative AI for text generation is summarised in this special issue of the *Canadian Journal of Cardiology*. Technologies like ChatGPT could, in the future, assist in writing administrative medical records and patient discharge summaries, thus streamlining administrative tasks and freeing cardiologists and other health professionals to focus on clinical duties. Recent developments have enabled generative AI models to focus on multimodal conversational AI for text or image generation, to create “general” multifaceted foundational medical models. In medicine, large language and vision assistants, such as LLaVA-Med,^114^ Med Palm 2, and the work by Zhu et al.,^115^ enable algorithms to perform various vision-centric tasks such as anatomic structure detection, segmentation, and disease classification, as well as language tasks such as report generation, open-ended question answering, longitudinal study comparison, and captioning a region of interest. These tasks can be performed on a multitude of data sets and provide a more holistic approach to test interpretation. In addition, generative adversarial networks are used to create realistic cardiac images and ECG tracings, help switch cardiac images from one modality to another, improve the quality of cardiac images, address missing data, and simulate patient responses to various therapies.^116,117^

In summary, the deployment of AI in acute cardiac care has the potential to revolutionise patient outcomes by offering rapid, precise diagnostics and effective management for CVDs, the leading global cause of death. However, harnessing AI’s full capabilities requires overcoming challenges such as data quality, bias, and transparency. Future integration of AI into clinical practice must involve careful regulation and continued innovation to maintain patient safety and adapt to evolving medical landscapes. With proper implementation, AI could become an invaluable asset in acute cardiac care, enhancing both the accuracy and the efficiency of patient treatment.

## Figures and Tables

**Table 1.** Performance and characteristics of automated algorithms for interpretation of electrocardiograms

Study PMID	Authors	Year	Outcome	Algorithm type	No. of ECGs (patients)	AUC	Sensitivity, %	Specificity, %	PPV, %	NPV, %

Conf erence paper	Fiorina et al.^23^	2023	Prediction of life-threatening ventricular arrhythmias	DL	78,294	0.911	83.3	88.7	—	—
37001583	Silva et al.^25^	2023	Acute pulmonary embolus	Ensemble neural network	1014	0.75	50	100	—	—
37386246	Al-Zaiti et al.^21^	2023	Risk stratification of occlusion MI	Tree SHAP algorithm RF	4026 (4026)	0.79	68.2	—	—	92.5
35987961	Choi et al.^22^	2022	HFrEF	DeepECG-HFrEF	18.449 (690)	0.844	77.9	76.3	70.8	82.4
34315566	Shrivastava et al.^26^	2021	Detection of dilated cardiomyopathy	AI-ECG: CNN model	16,446	0.955	98.8	44.8	—	—
32986471	Adedinsewo et al.^27^	2020	LV dysfunction in emergency department	AI-ECG CNN	(1606)	0.85	74.0	87.0	40.0	97.0
30821035	Attia et al.^28^	2019	LV systolic dyfunction (LVEF < 35%)	CNN	(16,056)	0.918	82.5	86.8	—	—
31074221	Kwon et al.^29^	2019	Heart failure identification (LVEF < 40%)	DL for ECG-based HF identification (DEHF)	55,163 (22,765)	0.843 internal 0.889 external	—	—	—	—
31525078	Tison et al.^30^	2019	Detection of hypertrophic cardiomyopathy	Combination of CNNs and hidden Markov models	36,186	0.91	—	—	—	—
31525078	Tison et al.^30^	2019	Detection of cardiac amyloid	Combination of CNNs and hidden Markov models	36,186	0.86	—	—	—	—

AI, artificial intelligence; AUC, area under the receiver operating characteristic curve; CNN, convolutional neural network; DL, deep learning; ECG, electrocardiogram; HF, heart failure; HFrEF, heart failure with reduced ejection fraction; LV, left ventricular; LVEF, left ventricular ejection fraction; MI, myocardial infarction; NPV, negative predictive value; PPV, positive predictive value; RF, random forest.

**Table 2.** Performance and characteristics of automated algorithms for interpretation of transthoracic echocardiograms

Study PMID	Authors	Year	Outcomes	Algorithm type	No. of studies (patients)	AUC	Sensitivity, %	Specificity, %	PPV, %	NPV, %

37611002	Holste et al.^39^	2023	Detection of severe aortic stenosis	3D CNN	6185 (2992)	0.978	85	96	—	—
34922865	Upton et al.^40^	2022	Severity of CAD	Automated image processing pipeline	578	0.93	92.7	84.4	—	—
35353118	Laumer et al.^41^	2022	Differentiation between AMI and takotsubo syndrome	Autoencoder model	(448)	0.79	—	—	—	—
31103590	Kusunose et al.^42^	2020	Detection of wall motion abnormalities	ResNet	(300)	0.99	—	—	—	—
32269341	Ouyang et al.^43^	2020	Prediction of HFrEF	3D convolutions with residual connection	7465	0.97	—	—	—	—
30354459	Zhang et al.^44^	2020	Diagnosis of HCM and cardiac amyloidosis	ResNet	14,035	HCM 0.93, cardiac amyloidosis 0.87	—	—	—	—

AMI, acute myocardial infarction; AUC, area under the receiver operating characteristic curve; CAD, coronary artery disease; CNN, convolutional neural network; EF, ejection fraction, HCM, hypertrophic cardiomyopathy, HFrEF, heart failure with reduced ejection fraction; NPV, negative predictive value; PPV, positive predictive value.

**Table 3.** Performance and characteristics of automated algorithms for interpretation of coronary angiography

Study PMID	Authors	Year	Outcomes	Algorithm type	No. of studies (patients)	AUC	Sensitivity, %	Specificity, %	PPV, %	NPV, %

37163297	Avram et al.^16^	2023	LVEF	DNN	4042 (3679)	0.906	77.9	88.6	66.0	93.4
37568050	Avram et al.^45^	2023	Coronary stenosis severity	CathAI (purpose-built neural networks)	13,843 (11,972)	0.862	74.5	78.1	46.1	92.4
35436189	Wu et al.^49^	2023	Coronary registration	DL	—	—	—	—	—	—
36265933	Park et al.^50^	2022	Plaque erosion	“Transformer-based” DL model	237,021 (581)	0.96	89.6	91.0	36.0	99.4
33213972	Moon et al.^51^	2021	Coronary stenosis	Key frame detection	452	0.956	—	—	—	—
33744790	Pang et al.^52^	2021	Coronary stenosis	Stenosis-DetNet (object detection networks)	166	—	82.2	—	—	—
34315031	Zhao et al.^53^	2021	Coronary stenosis	Feature pyramid with a U-Net++ model	314 (99)	—	0.86	1.00	68.4	—
32830647	Du et al.^54^	2021	Coronary stenosis and lesion morphology	Deep Discern—DNN	20,612	0.801	85.2	99.1	76.2	99.5
33528359	Chu et al.^55^	2021	Coronary plaque burden (OCT)	Deep convolutional network with encoding-decoding architecture and pseudo-3D input	509 (391)	—	—	—	—	—
33865741	Min et al.^56^	2021	Stent apposition (IVUS)	CNN, FNN	(618)	0.94	—	—	0.70	0.96
35003848	Yang et al.^57^	2021	Stent detection (OCT)	CNN	—	0.918–0.954	—	—	—	—
32034252	Lu et al.^58^	2020	Stent area (OCT)	Bagged detection tree	103	0.97	—	—	—	—
31978856	Ma et al.^59^	2020	Coronary roadmap	Bayesian filtering model	—	—	—	—	—	—
30426362	Jun et al.^60^	2019	Vulnerable coronary plaque (IVUS)	CNN, FNN, KNN	—	0.845	80.4	73.6	—	—

AUC, area under the receiver operating characteristic curve; CNN, convolutional neural network; DL, deep learning; DNN, deep neural network; FNN, feed-forward neural network; IVUS, intravascular ultrasound; KNN, K-nearest neighbuor; LVEF, left ventricular ejection fraction; OCT, optical coherence tomography; NPV, negative predictive value; PPV, positive predictive value.

**Table 4.** Performance and characteristics of automated algorithms for interpretation of cardiac computed tomography

Study PMID	Authors	Year	Outcomes	Algorithm type	No. of studies (patients)	AUC	Sensitivity, %	Specificity, %	PPV, %	NPV, %

37380806	Kesavuori et al.^72^	2023	Extraction of outer aortic surface in aortic dissection	3D CNN	206 (206)	—	—	—	—	—
35090845	Paul et al.^69^	2022	Coronary stenosis	Neural network composed of symmetric and asymmetric building blocks, including convolutions	(53)	—	93	97	—	97
32101464	Chen et al.^70^	2020	Coronary stenosis	DL	(124)	0.87	94	63	93	73
33778579	Martin et al. ^71^	2020	AI-based CT-FFRin triple-rule-out in acute chest pain	cFFR	159 (159)	—	—	—	—	—
26158081	Kang et al. ^73^	2015	Coronary stenosis	SVM	42	—	93	95	—	—
22484719	Goldenberg et al.^74^	2012	Coronary stenosis	CAST	2000	—	> 90	40–70	—	> 95
19890640	Arnoldi et al.^75^	2010	Coronary stenosis	Automated detection algorithm	59	—	100	65	58	100
22003680	Kelm et al.^76^	2011	Coronary stenosis	Supervised learning	229	—	97.62	67.14	—	99.77

AUC, area under the receiver operating characteristic curve; CAST, computer-aided simple triage; CNN, convolutional neural network; CT, computed tomography; DL, deep learning; FFR, fractional flow reserve; SVM, support vector machine; NPV, negative predictive value; PPV, positive predictive value.

**Table 5.** Performance and characteristics of automated algorithms for interpretation of cardiac magnetic resonance imaging

Study PMID	Authors	Year	Outcomes	Algorithm type	No. of data sets (patients)	Accuracy	AUC	Sensitivity, %	Specificity, %	PPV, %	NPV, %

30084736	Baessler et al.^84^	2018	Detection of myocarditis	Texture analysis	(39)	—	0.88	89	92	—	—
36943075	Ghanbari et al.^85^	2023	Scar analysis and arrhythmia prediction	CNN Ternaus network	591 (761)	—	0.67	—	—	—	—
36124774	Zhang et al.^44^	2022	Scar assessment in MI	DL virtual native enhancement	4721 (912)	—	—	77	100	—	—
35240789	Sharifrazi et al.^86^	2022	Detection of myocarditis	CNN + K-means clustering	98,898	—	0.97	—	98.6	—	—
—	Moccia et al.^87^	2020	Scar detection	Local binary patterns, spatiotemporal features	328	—	0.75	—	—	—	—
36713008	Alabed et al.^88^		Mortality prediction in newly diagnosed PAH	Multilinear principal component analysis	723 (737)	70	—	—	—	—	—

AUC, area under the receiver operating characteristic curve; CNN, convoluted neural network; DL, deep learning; MI, myocardial infarction; PAH, pulmonary arterial hypertension; NPV, negative predictive value; PPV, positive predictive value.
